# P4 Medicine as a model for precision periodontal care

**DOI:** 10.1007/s00784-022-04469-y

**Published:** 2022-03-28

**Authors:** P. Mark Bartold, Sašo Ivanovski

**Affiliations:** University of Queensland, 1 Milton Avenue, Beaumont, South Australia 5066 Australia

**Keywords:** P4 Medicine, Periodontics, Patient management

## Abstract

**Objectives:**

P4 Medicine is based on a proactive approach for clinical patient care incorporating the four “pillars” of prediction, prevention, personalization, and participation for patient management. The purpose of this review is to demonstrate how the concepts of P4 medicine can be incorporated into the management of periodontal diseases (particularly periodontitis) termed P4 periodontics.

**Methods:**

This is a narrative review that used current literature to explore how P4 periodontics can be aligned with the 2018 Classification of Periodontal Diseases, current periodontal treatment paradigms, and periodontal regenerative technologies.

**Results:**

The proposed model of P4 periodontics is highly aligned with the 2018 Classification of Periodontal Diseases and represents a logical extension of this classification into treatment paradigms. Each stage of periodontitis can be related to a holistic approach to clinical management. The role of “big data” in future P4 periodontics is discussed and the concepts of a treat-to-target focus for treatment outcomes are proposed as part of personalized periodontics. Personalized regenerative and rejuvenative periodontal therapies will refocus our thinking from risk management to regenerative solutions to manage the effects of disease and aging.

**Conclusions:**

P4 Periodontics allows us to focus not only on early prevention and intervention but also allow for personalized late-stage reversal of the disease trajectory and the use of personalized regenerative procedures to reconstruct damaged tissues and restore them to health.

**Clinical Significance:**

P4 Periodontics is a novel means of viewing a holistic, integrative, and proactive approach to periodontal treatment.

## Introduction

The term “P4 medicine” was coined by Dr. Leroy Hood and has been hailed as the "future of medicine" [1–3]. The basis of this concept is that medicine should move from being a reactive to a proactive clinical approach for patient care. Thus, a wellness model has evolved whereby the objective of patient management is to focus on overall patient well-being rather than merely treat a disease and its symptoms. Within this model, participation, prediction, prevention, and personalization form the basis of the 4Ps for patient management (Table 1). P4 Medicine embraces a new paradigm of holistic and integrative practices for patient management with an equal participation between patient and practitioner for overall health care. Combining precision medicine concepts and whole life medical experiences, it is possible to incorporate novel diagnostics, risk measurements, patient participation, and technological advancements to improve all aspects of healthcare delivery.

**Table 1:** P4 Medicine^*^

Predictive	To be able to predict the potential future emergence of disease-perturbed networks in patients
Preventive	To design “preventive drugs” that will block the emergence of these disease-perturbed networks and their cognate diseases
Personalized	To treat each person as a unique individual and not as a statistical average
Participatory	To rely greatly on the positive contributions of activated patients and consumers

^*^P4 Medicine is a concept that proposes a holistic and integrative approach to health and disease, empowering patients to actively participate in the improvement of their own healthcare

In this review/commentary, we discuss how the new classification of periodontal diseases is consistent with, and complimentary to, the incorporation of P4 medicine into the management of the periodontal diseases (particularly periodontitis). For this discussion, we will limit our focus to individual patient care. Nonetheless, it is important to acknowledge that the P4 medicine concept is greater than the individual and encompasses the whole spectrum of health and well-being of the macro-environment of global populations and communities to microlevels of individual body systems (Table 2).

**Table 2:** P4 Medicine intervention levels

Global and Region Levels
Communities within Regions
Individuals within Regions
Systems within Individuals

## Reimagining holistic oral health management

It is well accepted that oral health is a very significant public health issue. Current epidemiological data indicate that around 50% of the global population presents with dental caries, various forms of periodontal disease, and loss of teeth [4]. Of concern is that notwithstanding significant research into understanding the pathogenesis of dental caries and periodontal disease as well as their prevention, this incidence of oral disease has not changed over the past 25 years. Although the World Dental Federation (FDI) set a target to improve overall oral health by 2020 [5], this appears to have been an ambitious goal. Clearly the dental profession should reconsider its current approaches for the management of all chronic and long-standing oral bacterial infections. This review focuses on the plaque-associated periodontal diseases and how current advances are changing the paradigms for the management these conditions.

## Periodontal health and disease reappraised

A revised classification of the plaque-associated periodontal diseases was presented in 2018 [6]. This highlighted that the plaque-associated periodontal diseases were most likely a “spectrum” of conditions that ranged from periodontal health to terminal and dentition-threatening disease [6]. Importantly the baseline condition of “periodontal health” was addressed. It was indeed surprising that until 2018 the classification of periodontal health had not been definitively described. Now that periodontal health has been clearly defined, the periodontal diseases can be classified through a sequential process of stage and grade allocation.

Whole life health and well-being is now a very important focus and goal for a large proportion of the population. To achieve this, emphasis is placed on holistic well-being, the early interception of chronic disease, and management of modifiable risk factors. In this context, periodontal management should include processes to describe the transition phases of periodontitis over a life course and determine strategies to revert the disease path toward health as early as possible. Utilizing the principles of P4 medicine, reversion of disease will not be driven by practitioners working in isolation but rather will require an integrative and inclusive approach utilizing a “multisector approach” engaging not only clinical acumen but also advances in the integration of systems biology and associated technological advances. To achieve the goal of changing transition pathways from disease trajectories to health acquisition, there is a requirement for clear guidelines that characterize the various stages of health and disease (gingivitis and periodontitis), as well as an understanding of the levels of intervention that are needed to drive the transitions toward health. The stages of health, gingivitis, and periodontitis have been addressed recently to provide a framework for diagnosis and management [7, 8].

**Fig. 1:**
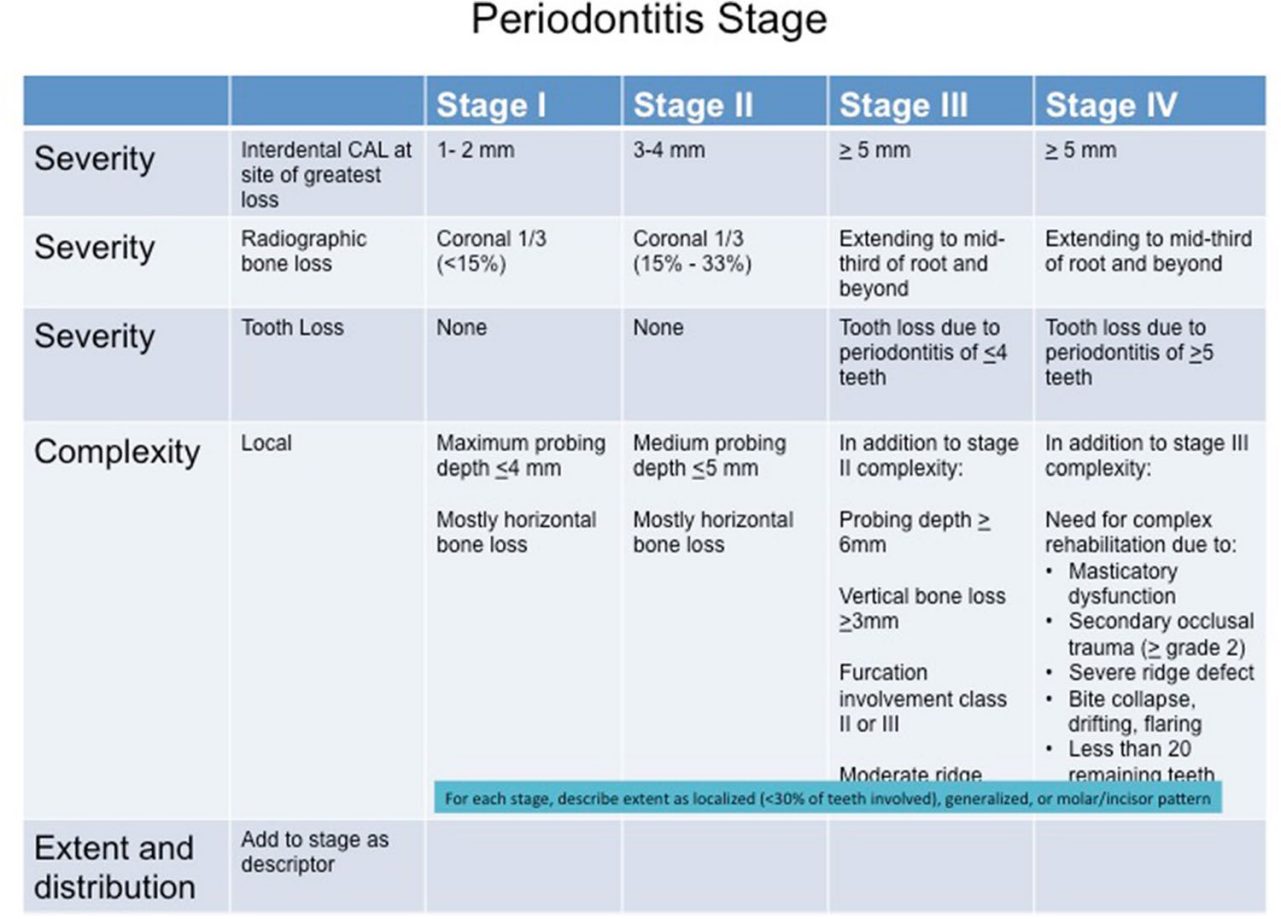
Stages of plaque-associated periodontal disease. Adapted, with permission from publisher, from Papapanou et al. [8]

**Fig. 2:**
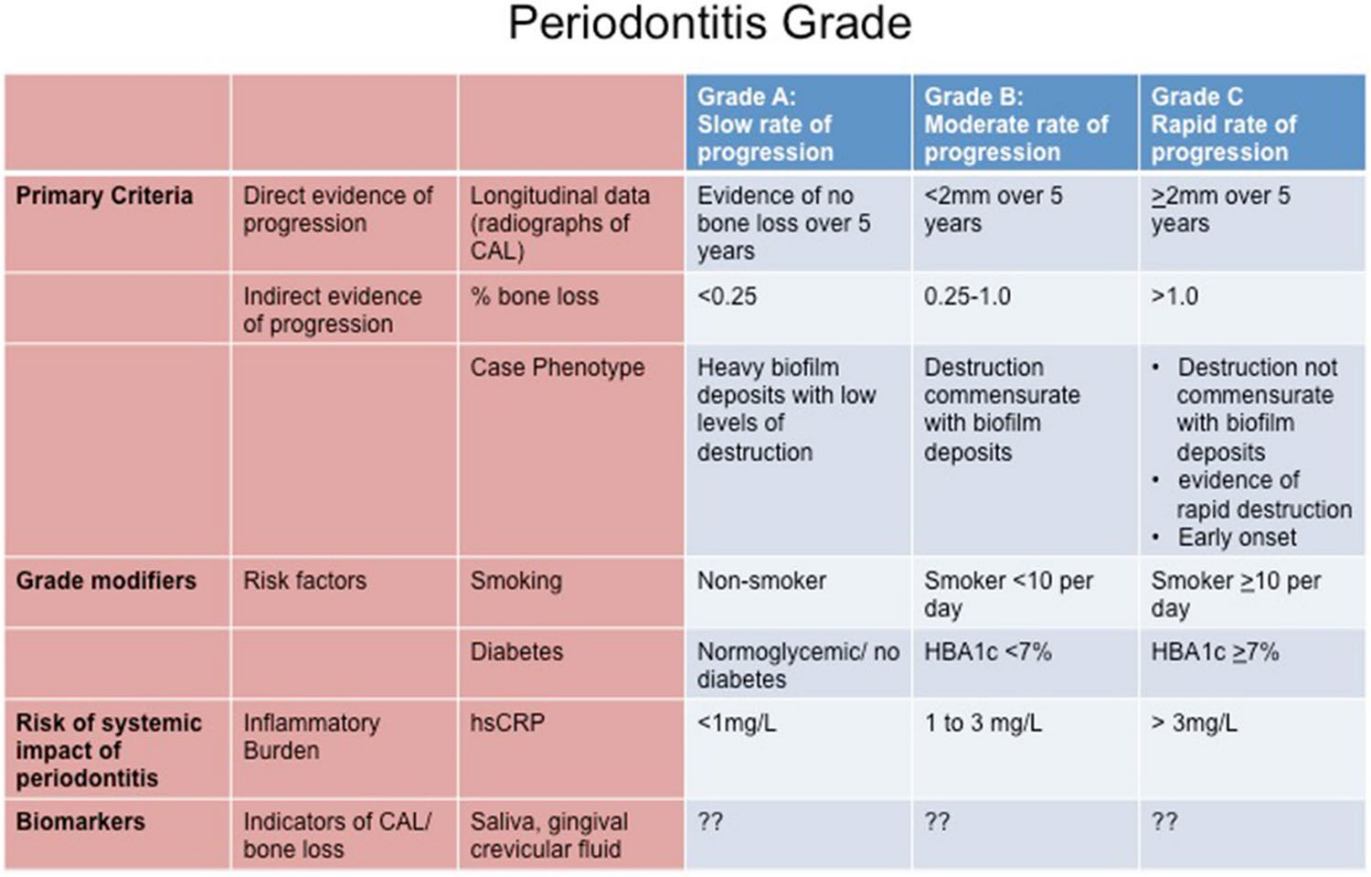
Grades of plaque-associated periodontal disease. Adapted, with permission from publisher, from Papapanou et al. [8]

Although not immediately apparent at the time, the 2018 revised classification of periodontal diseases is consistent with the concepts of P4 medicine because of the way that the plaque-associated periodontal diseases are classified into stages and grades according to severity, rate of progression, and effect of modifying risk factors (Figs. 1 and 2) [8, 9]. The pathway that a disease follows is usually correlated with the rate of disease progression relative to patient age. This so-called trajectory of the disease can then be graded as low, moderate, or rapid (Fig. 2). This grading of periodontitis embraces many aspects of the P4 medicine model because it focuses on the predictive, preventive, and personalized components of the life course of periodontitis. Although the participatory component of P4 medicine is missing from this classification model, it is not difficult to incorporate this into a P4 periodontics model. Indeed, allowance for an individual to actively participate in their periodontal health care is important for conveying ownership of the problem. This has the potential to positively impact on the control and management of an individual’s periodontal disease.

An important factor in the concepts of P4 medicine is recognizing that disease development and progression trajectories can be partially attributed to a change in allostasis (a state of equilibrium) [10]. As disease develops, there is a loss of homeostasis due to an ever-increasing allostatic load. Allostasis is defined as the process of maintaining homeostasis through the adaptive change of the organism’s internal environment to meet perceived and anticipated demands [11]. Allostatic load is the result of cumulative increases in internal and external stressors including physiologic, environmental, and lifestyle stresses that combine negatively to impact to the mediators of allostasis. Many common chronic diseases, including gingivitis and periodontitis, can be considered to be a consequence of allostatic overload [12]. Thus, in order for gingivitis to develop, there must be a shift in allostatic load consistent with health (homeostasis) to allostatic overload (imbalance) and resultant disease. If further allostatic dysregulation occurs, periodontitis will develop.

**Fig. 3:**
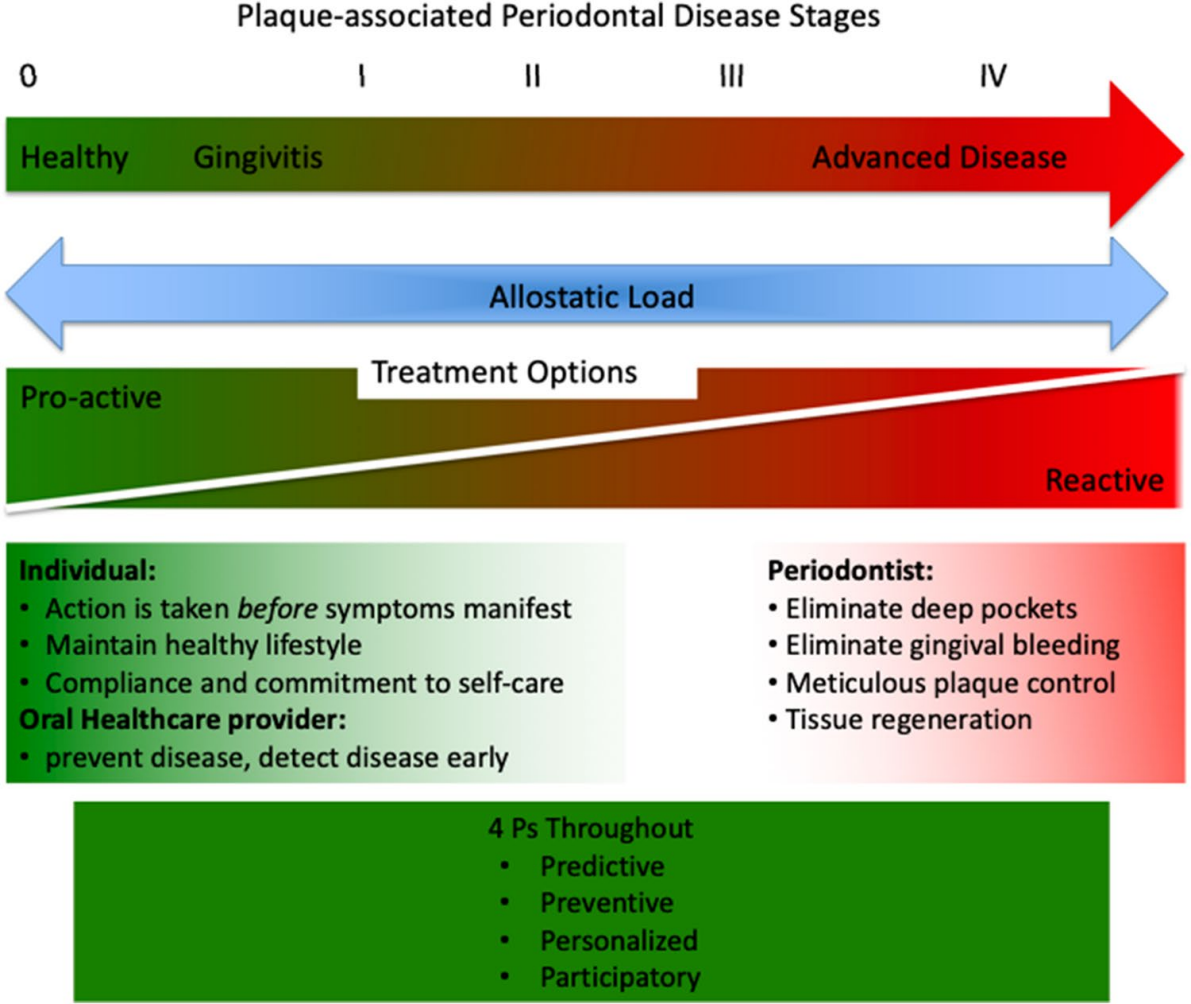
Incorporation of P4 Periodontics into the classification of plaque-induced periodontal disease

Disease management within the P4 Periodontics continuum can be either proactive or reactive depending on the patient or periodontist-mediated treatment strategies. In this context, the influence of allostatic load (both internal and external stressors) may be beyond an individual’s self-control and thus require treatment intervention (Fig. 3). In keeping with the 2018 classification, the stages and grades of periodontal disease can, in part, be attributed to allostatic load and embrace the concepts of P4 medicine.

## Stage classification of periodontitis

### Stage 0—periodontal health—allostasis or general homeostasis

Periodontal health has been defined as the absence of clinical signs and symptoms of gingivitis or periodontitis allowing normal function and no deleterious mental or physical effects associated with past damage caused by periodontal inflammation [13]. From this definition, periodontal health can exist within four different scenarios that are related to whether the periodontium is structurally and clinically sound or affected by previous disease. These have been classified as (1) pristine periodontal health in a structurally sound and uninflamed periodontium, (2) well-maintained clinical periodontal health on a structurally and clinically sound (intact) periodontium, (3) periodontal disease stability on a reduced periodontium, and (4) periodontal disease remission/control on a reduced periodontium [7]. Thus, periodontal health can be identified either as a condition that has not experienced any active disease or as a condition that has previously experienced disease activity and associated tissue damage but has reverted back to a state of health and absence of active inflammation. The latter condition of periodontal health generally arises as a result of periodontal treatment that has reduced the infection and inflammation to a level that is compatible and consistent with periodontal health. This reversion of disease results in periodontal disease stability or remission on a reduced periodontium. Within the P4 periodontics model, periodontal health is a principal goal. This requires continuous monitoring to control the development of adverse events particularly those associated with uncontrolled risk factors, modifying factors, and allostatic load.

### Gingivitis—emergence of allostatic imbalance

As the most prevalent form of periodontal inflammation, gingivitis is a recognized risk factor for subsequent destructive periodontitis. According to the 2018 Classification of Periodontal Diseases, gingivitis is a well-defined periodontal disease entity with several different clinical manifestations and associations with systemic risk and aggravation factors [7]. Gingivitis can be defined as a non-specific inflammatory response to a non-specific accumulation of dental plaque. This condition is reversible following adoption of good oral hygiene practices and significant reduction in plaque load.

### Stage I—initial (early/mild) periodontitis (allostatic load increases)

Stage I initial periodontitis will usually be associated with clinical signs and symptoms of gingivitis together with early-stage damage to the periodontium including initial (up to 15%) coronal bone loss. At this point, patients may not be aware of this initial stage of disease and almost certainly do not experience any functional difficulties. If left untreated, the risk for further disease progression is high.

### Stage II—moderate periodontitis (initial exposure to changing allostatic load)

Stage II moderate periodontitis is characterized by continuing gingival inflammation and associated loss of periodontal attachment and coronal bone loss up to one third of root surfaces. At this stage, probing periodontal pocket depths up to 5 mm may be recorded and a general pattern of horizontal bone resorption is noted. The effects of various predisposing and modifying stressors that can influence the development of clinical signs and symptoms of periodontitis begin to influence the rates of disease progression. While there may be some signs and symptoms of periodontal inflammation (bleeding gingiva following toothbrushing and flossing), many patients will still be unaware of any significant periodontal problem at this stage. Thus, unless the periodontitis is detected at this stage by an oral health professional, the likelihood of treatment is low. If detected, and treatment is provided, it is usually possible to alleviate the symptoms.

### Stage III—severe periodontitis with potential for additional tooth loss (increasing allostatic load)

Stage III severe periodontitis is characterized by pocket depths greater than 6 mm, vertical bone loss encroaching the middle third of root surfaces, and advanced (grade II/III) molar furcation involvement. Loss of teeth due to the periodontal damage may occur but is limited to no more than 4 teeth. Treatment provided at this stage targets the disease signs and symptoms and is reactive to the general extent of the disease. This treatment strategy is often limited to managing the clinical features of periodontitis with little focus on the underlying driving allostatic forces complicating disease manifestation such as unhealthy lifestyle, systemic conditions, external stressors, and predisposing and modifying factors.

### Stage IV—advanced periodontitis with extensive tooth loss and potential for loss of dentition (complete allostatic overload)

Stage IV advanced periodontitis, the final stage of the disease, can be considered terminal and requires complex reactive and rehabilitative treatments. In addition to the features noted for stage III periodontitis addition, complex issues arise due to advanced loss of periodontal support, associated advanced bone loss into the apical third of root surfaces, and loss of more than 5 teeth with associated functional difficulties. Treatments can be provided that generally target the extent and severity of the disease and will also require advanced prosthodontic solutions to enable restoration of reasonable comfort and function for the patient. At this stage of disease expression, the allostatic load has most likely progressed to “overload.”

## Limitations of current periodontal therapy

The staging and grading classification of periodontitis take into account our understanding that patients present with varying combinations of clinical signs, symptoms, risk factors, and modifying factors. Clearly, these features dictate that treatments should target these aspects. However, this is not always the case and reactive, rather than proactive, treatments are the principal focus of traditional periodontal therapy. This reactive approach includes a focus on meticulous plaque control, reduction of pocket depths, and elimination of gingival bleeding. Unfortunately, this approach does not always lead to the desired outcomes. As a result, this focus on anti-infective therapy and reduction of tissue damage has been questioned [14]. Indeed, it may be that such generic approaches to periodontal treatment are unsuccessful in up to 20% of patients with periodontitis [15–18], often those that are most susceptible to the disease. Thus, by applying generic management protocols to all patients is flawed because this approach requires the management of an “average” rather than the individual patient [19]. While this “one size fits all” strategy can be very successful in some individuals, it is not always the case. Thus, contemporary periodontal treatment should not consider the “average patient” but rather consider the individual patient in the context of the intricacies of human biology, allostasis, and genetic control of the host response to external stressors.

## Can periodontitis stage status change?

Without treatment any reversal of periodontitis from one stage to another is very unlikely. Spontaneous regression of disease may occur but only while in the initial stage, and this would require a very significant effort on the patient’s behalf to improve oral hygiene and control any other mitigating factors. Once the more advance levels of stage II and III periodontitis have developed, reversion to periodontal health is an important goal but not always achieved. Nonetheless, for patients with stage II or III disease, it is possible that with concurrent reactive periodontal therapy (including regenerative procedures) and necessary lifestyle changes, a patient could return to periodontal health. In these cases, health would be either associated with a reduced periodontium or disease remission [13]. Thus, by applying the principles of P4 medicine (prevention, participation, prediction, and personalization), it is possible to return a patient to periodontal health from periodontitis stages I–III, but not stage IV due to the extensive tooth loss associated with this stage that necessitates extensive prosthetic reconstruction.

## P4 Periodontics—a new management paradigm

As detailed above, reactive periodontal treatments do not embrace a truly holistic approach to patient management. Accordingly, it is important to explore new models as contemporary vehicles for the delivery of health care and management of chronic diseases [2, 3]. P4 Medicine has thus evolved to provide multilevel health care model for management of chronic disease. Since periodontitis is one of the most common chronic diseases affecting humans, the term “P4 periodontics” has been proposed [20].

Incorporation of P4 periodontics into management strategies will require consideration of the four fundamental aspects central to a personalized approach and patient stratification for the management of all chronic diseases. These are disease severity, disease activity, disease control, and response to treatment. At present, our capability to address these issues is limited [14]. However, it is expected that currently evolving technologies will allow rapid advancements in each of these critical areas of disease assessment.

### Disease severity

The determination of disease severity is important because it determines the required levels of treatment intervention. The tools and methods used to measure severity of periodontitis have not changed in decades. The principal methods used include visual assessment of bleeding following probing, crude measurement of pocket depth, and loss of attachment using a periodontal probe, imprecise measurement of tooth mobility and furcation involvement, and two-dimensional radiography. In order to advance, periodontology is in need of more sophisticated methodologies to assess the severity of tissue damage arising from periodontitis. There are many areas that offer considerable potential, and these include advanced radiographic imaging, real-time microbiome assessment, proteomic profiling of gingival punch biopsies, and assessment of disease biomarkers in blood, saliva, and gingival crevicular fluid. The identification of disease severity is central to the initial staging in the 2018 periodontitis classification.

### Disease activity

A critical aspect in the management of periodontitis should be a means to determine whether the disease is currently active or in a phase of inactivity or remission. This will allow different management protocols depending on whether or not the disease is undergoing a current phase of activity and associated continuing tissue destruction. However, current diagnostic tools to detect disease activity are severely limited in their capacity to distinguish disease active from disease in-active sites.

### Disease control

Current strategies to control periodontitis are based on an understanding that it is an opportunistic infection modified by host inflammatory response. Thus, addressing the effects of both inflammation and infection as well as identifying predisposing and modifying factors associated with disease development and progression are important. Once stabilized, the disease can be further controlled through regular and ongoing re-assessment and supportive periodontal care [21]. As detailed above, there is a lack of well-validated biomarkers for disease activity or severity. Clinicians are still restricted by the relatively crude objective markers for disease control such as recording improved plaque control, reduction in bleeding on probing, reduced probing pocket depths, and gain of clinical attachment. This deficiency in development and utilization of sophisticated outcome measures negatively impacts on our ability to determine the level of disease control achieved following active periodontal treatment in a subjective and quantitative manner.

### Response to treatment

Like all chronic diseases, the responses to conventional periodontal treatment can be variable. Indeed, a number of studies have reported that approximately 20% of periodontitis patients may have a less than adequate treatment outcome [14–16]. The remaining 80% of periodontitis patients can be expected to respond favorably to treatment, but it should be noted that this can be to varying extent and at variable rates. Determining the unique response of individual patients to periodontal treatment will be an important component of personalized periodontics. Factors such as individual’s microbiome, inflammasome, and allostatic load will all impact on predicted outcomes.

## Integration of P4 periodontics into holistic periodontal management

A coordinated patient-centered and systems-based approach for management of chronic disease forms the basis of P4 medicine. Its goal is to be able to predict and forecast disease susceptibility in healthy individuals well before clinical manifestation of disease occurs. This approach aims to optimize individual health and avoid onset of disease. It involves the collection and analysis of data collected via diagnostic tests, mobile health apps, and other tools that can assist people understand and recognize individual risk factors and how to control them. An overarching requirement is that all of these processes are based on sound evidence-based information to guide overall treatment goals and expected outcomes [22].

**Fig. 4:**
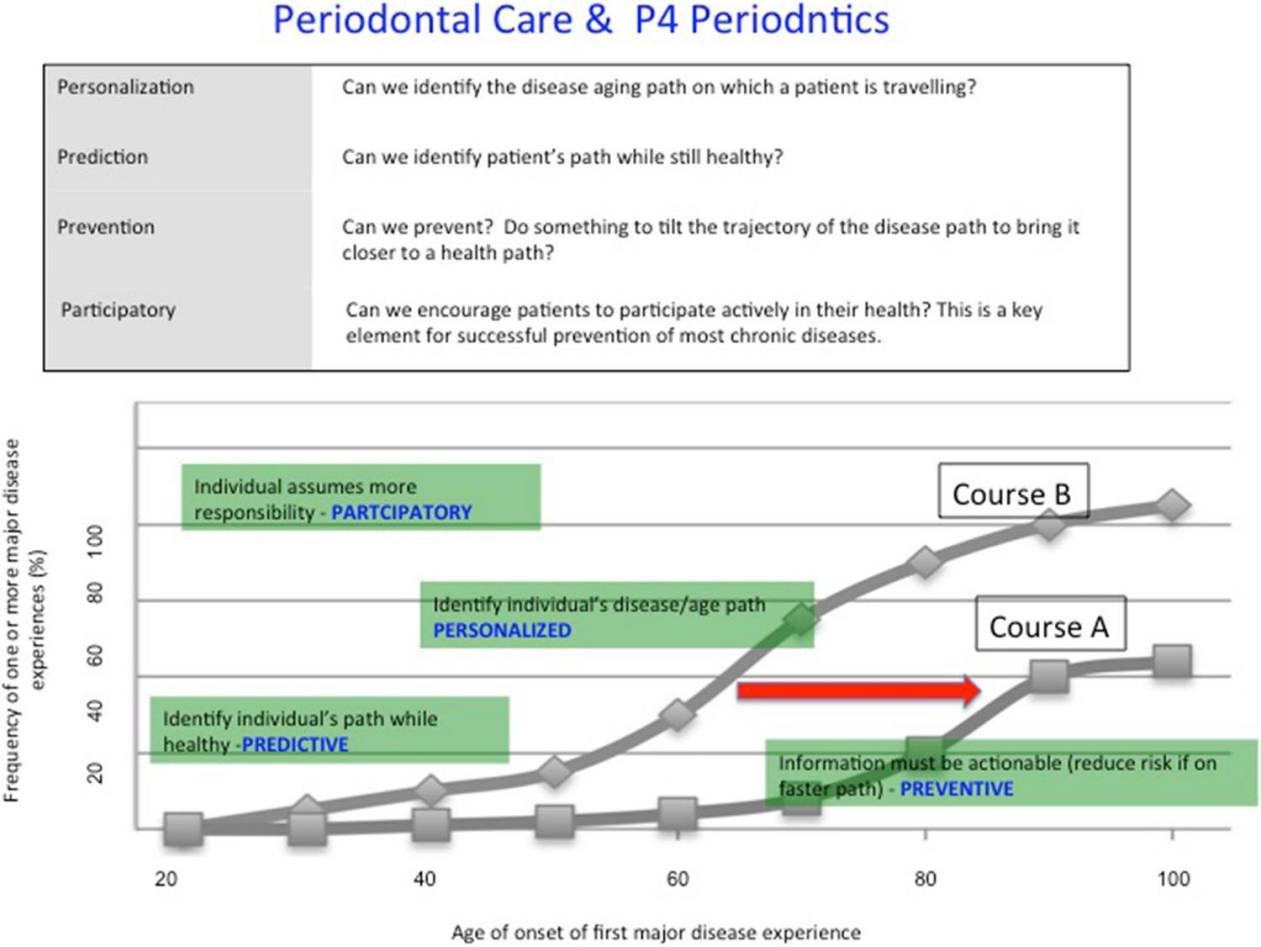
Periodontal care and P4 periodontics. Incorporation of the principles of P4 medicine into periodontics. In this model, the management of plaque-associated periodontal disease can incorporate all of the elements of P4 medicine: personalization, prediction, prevention, and participation. Adapted, with permission from publisher, from Kornman et al. [22]

Plaque-induced periodontitis has all of the hallmarks of a chronic disease and as such the adoption of P4 medicine principles into periodontology is attractive. Management of plaque-associated periodontitis through each of its life-course stages can incorporate all of the elements of P4 medicine: personalization, prediction, prevention, and participation (Fig. 4). In this context, two commonly encountered trajectories of disease experience over a lifespan may be considered. These are either a slowly progressing form resulting in mild to moderate periodontitis over a lifetime or a more aggressive and rapidly advancing form of periodontitis resulting in very significant periodontal damage over a lifetime [23]. By applying the P4 principals of prediction, prevention, personalization, and individual participation in disease control, it is apparent that the more aggressive life course can be manipulated toward a more moderate life course.

### Predictive periodontics

**Fig. 5:**
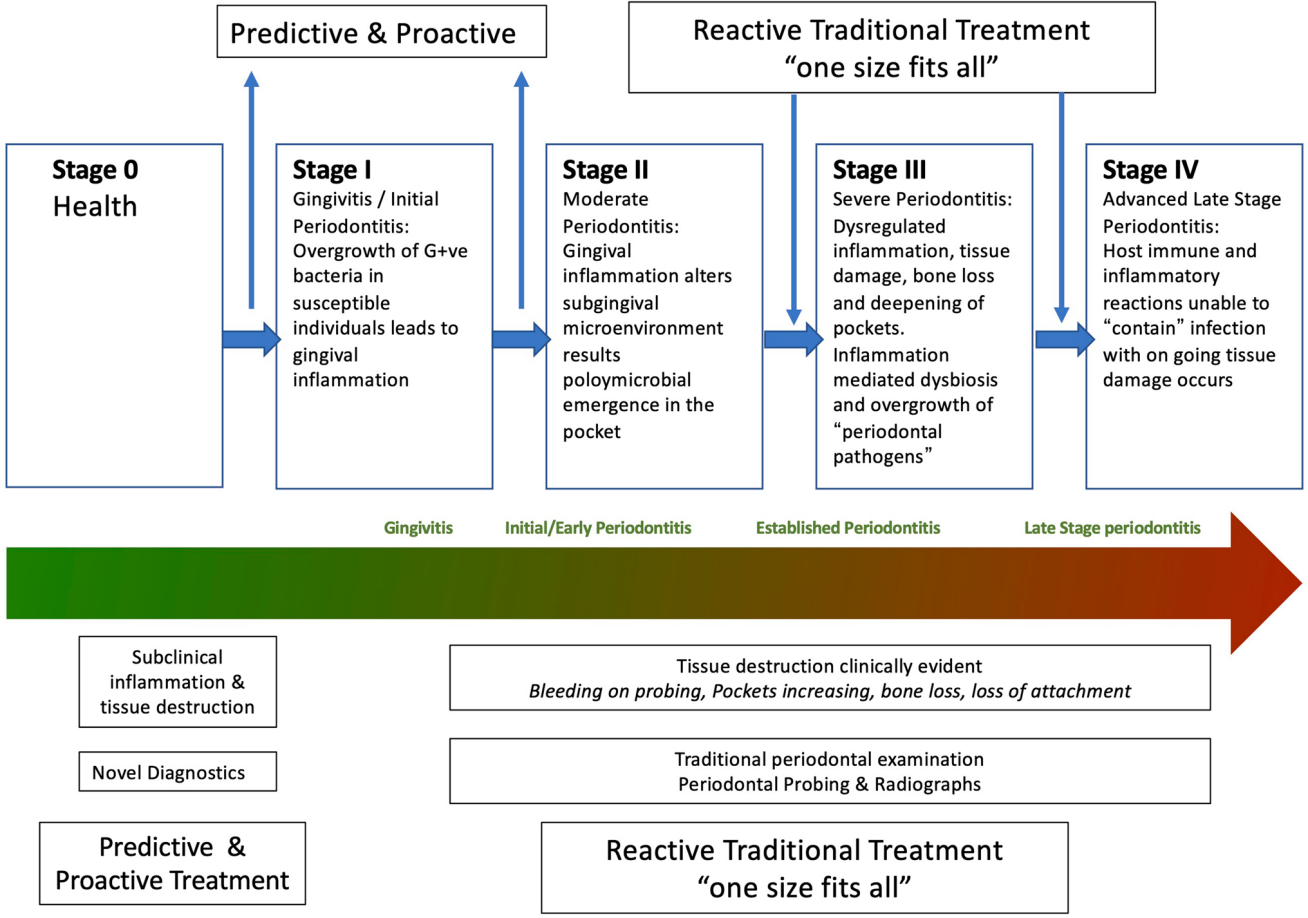
The basis of this concept is that medicine should move from being a reactive to a proactive clinical approach for patient care

Through understanding the molecular basis of disease, systems medicine should be able to predict the likelihood of an organ becoming diseased or when a disruption to a specific biological network would result in disease. Accordingly, identification of factors that represent precursor elements to the clinical presentation of stage I periodontitis would allow intervention strategies based on the control of initial disease pathways. As a result, proactive (rather than reactive) preventive measures can be adopted (Fig. 5). Prediction of the risk of future disease development is a very important aspect of preventive medicine and focuses on the identification of risk factor phenotype prediction.

To incorporate the P4 concepts into prediction for periodontal disease, established risk factors such as poorly controlled diabetes and smoking will need to be considered in conjunction with contemporary risk assessment that could include microbiome, inflammasome, epigenetics, and allostatic load analyses. The utilization of molecular diagnostics developed from blood, saliva, and gingival crevicular fluid is central to this. This information will be obtained from a variety of healthcare settings allowing the input of data that can be collated in data management systems to develop algorithms that assist in identifying individuals at risk of developing disease and capturing this before disease occurs.

### Preventive periodontics

Prevention of development of periodontal disease has been a long-standing goal in periodontology. It has been based on a flawed approach of focusing almost entirely on meticulous plaque control rather than recognizing individual variability in host responses to dental plaque. For decades, we have recognized that some people can tolerate quite heavy plaque deposit yet manifest very little disease while others over-respond to even the slightest amount of plaque, resulting in severe and difficult-to-control periodontitis [24–27]. Thus, the effectiveness of oral hygiene on the incidence and management of advanced periodontitis in various communities has been questioned [28, 29]. For example, it has been noted that the prevalence of severe periodontitis has remained static [30] despite the widespread use of oral hygiene aids, persistent reinforcement of the need for meticulous oral hygiene by the dental profession, and the associated general improvement in oral hygiene levels in the community [29]. Furthermore, it has been observed that although improved oral hygiene practices, devices, and products have a positive effect as manifested by a reduction in plaque scores and in the prevalence of gingivitis, they have had little effect on the prevalence of periodontitis [28].

Accordingly, prevention of plaque-associated periodontitis requires a reassessment of traditional plaque-focused programs and embracing our contemporary knowledge regarding the etiopathogenesis of periodontitis. Indeed, the relationship between the periodontal biofilm and the host response in the pathogenesis and management of periodontitis is being viewed with renewed interest and focus [31, 32]. Accordingly, a model has been presented that focuses attention on the central role of inflammation and how this might control the specific infections associated with periodontitis [33–35]. Progress is being made in integrating etiopathogenesis into preventive concepts through investigating important biological system networks, how allostatic load and risk factors might influence these, and identification of endogenous protective factors that might help to prevent disease establishment or progression. In the context of periodontitis stages, it may be possible to change disease pathways back toward health. Indeed, by preventing disease progression at stages 0 and I, it may be possible to negate or dampen the effects of adverse biologic network systems to prevent or reverse early periodontitis. By considering the nexus between periodontal inflammation and infection, a simple model has been presented in the context of controlling inflammation to control infection and thus drive gingivitis back to health [33, 36].

### Personalized periodontics

It is well understood that patients manifest periodontitis in variable patterns associated with specific clinical signs, symptoms, risk factors, and modifying factors. This variability dictates that treatment should embrace and account for these factors. However, this is not always the case and, as detailed earlier, generic rather than patient-specific treatments remain the foundation of traditional periodontal therapy. However, this generic periodontal therapy is based on flawed assumptions that are biased toward managing the “average” patient in a “one size fits all” approach. This generic approach can produce good treatment outcomes in some patients, but not all. More recently the concept of patient stratification has been presented as a central part of personalized periodontics [23].

Patient stratification for periodontitis is a process that recognizes this complex disease arises via multiple pathways impacted by multiple risk factors that can influence that condition either in their own right or in an additive manner. Thus, risk factors need to be integrated at the individual patient level. This can be achieved by focusing on four core questions:

1. What are an *individual’s* major risk factors for developing periodontitis?
2. Can *individual* risk for developing periodontitis be identified?
3. Can *individual* risk for periodontitis progression be identified?
4. Can *individual* treatment response/outcomes be determined?

For personalized periodontics to be successful, it will be critical to be able to determine those individuals who are unlikely to respond to generic (conventional) treatments. These “non-responder” patients will require far more sophisticated diagnostic approaches and treatment goals. These are the individuals for whom the periodontal condition is poorly correlated with the amount and length of exposure to dental plaque. For these individuals, risk factor modulation of host immune and inflammatory responses most likely dictate the level of disease progression and severity. More recently, an understanding of the microbiome and its relevance to antibiotic resistance through the resistome (resistance capabilities of bacteria preventing the effectiveness of antibiotics) has been recognized as being of considerable importance to a personalized approach for disease management [37, 38]. By identifying these individuals and their specific bio-, pheno-, and genotype individual and personalized traits can be characterized to assist in controlling the course of their periodontitis disease experience. In order to achieve this, disease systems and biology networks will be required incorporating network communication processes to access individual genome, microbiome, resistome, and inflammasome data. Once these data are collected, they can be utilized through all four levels of the P4 periodontics concept.

### Participatory periodontics

Empowering patients to take an active role in their periodontal health has always been an important part of integrated periodontal treatment. However, like other aspects of periodontal treatment, this is not always successful. Patients newly diagnosed with periodontitis will invariably inquire about why they have developed this condition, what did they do wrong, and what can they do about it once it has been diagnosed. This indicates that patients want some ownership of their problem but need guidance. The response usually is one of generic oral hygiene instruction and “motivation” and possibly addressing lifestyle issues that can impact on the disease (e.g., control of smoking and diabetes). Unfortunately, despite patients’ desire to participate in their periodontal care, this approach is not always successful due to lack of individualization of the information and instruction provided [39].

It is well accepted that patients must participate in their treatment that incorporates a preventive, predictive, and personalized approach. Thus, participatory periodontics will require a patient-centered embracement of “oral health literacy.” Health literacy is the process through which patients acquire and use simple health information and support services to facilitate their own health management [40].

One way that patients can obtain base level health information is through self-tracking devices that allow them to monitor individual health-related data. In recent years, there has been very significant and rapid development of technology to monitor health-related data through mobile applications or “apps.” This information is known as patient-reported experience measures (PREMs) and is designed to assist patients in collecting and reporting real-time data recording of their health information and quality of life experiences. This information can be fed back into databases and linked to an individual’s health records for future use in their overall health management plan. This process will assist healthcare workers to develop a personal level of interaction and delivery of personalized care via individual targeted goals and healthcare plans based on an individual’s self-generated health profile.

This technology can also assist users change their behavior to achieve specific goals to manage their health and well-being [41]. For example, using this technology, individuals can self-monitor sleep patterns, posture status, nutrition, blood glucose levels, and even oral health practices and status. It is anticipated that medical health apps will continue to evolve and allow individuals personalized 24-h healthcare access, services, and feedback at relatively low costs [42]. There are many oral health apps available, including smart toothbrushes connected to various phone apps, that bring the dentist to the patient virtually. These assist in monitoring in real time how teeth are brushed, what sites have been missed, and assistance in setting oral hygiene goals. However, these developments are in their infancy and it has been concluded that many of these are of relatively poor quality. Hence, there is a need for further development of apps that have evidence-based content, have been appropriately validated, and are user friendly to allow easy access to quality oral health data management [43].

Participatory periodontics is aligned with the concept of collating and providing timely health information for patients and oral health practitioners. With the increasing trend of patients wanting to be involved in the management of their health conditions, the participatory aspect of P4 periodontics presents important new pathways to enable patient access to, and participation in, their periodontal care. In the P4 periodontics model, patient participation recognizes the importance of new modes of transfer and utilization of information between patients and their healthcare provider for all stages of periodontal health and disease.

## The big picture

As detailed above, P4 periodontics will progress periodontology from being a reactive discipline to a proactive discipline incorporating the 4Ps: predictive, personalized, preventive, and participatory. Of course, there are still many technical and social issues that need to be considered before this approach becomes reality. New strategic partnerships between dentistry, patients, health service providers, and health insurance agencies will need to be forged to collect and collate the data needed to predict and manage disease before it occurs. Rapid developments in the fields of next-generation sequencing, proteomics, genomics, and biosensing have resulted in a rapid expansion of biomedical big data.

The new concept of personalized and actionable health care dictates the use of enormous amounts of data associated with myriad dimensions of personal health and associated systems analytics. Enormous volumes of biomedical data can be amassed from a variety of sources including patients’ self-reporting. Patients’ biological data, health research, electronic health records, personal health, and fitness recording devices and even social media. Thus, both healthy and sick people will be providing masses of assessable data concerning their health and disease. Potentially, these data will assist in identifying potential risk factors, unknown links with other diseases and permit evaluation of treatment or intervention outcomes. These data will need to be depersonalized, aggregated, stratified, and analyzed to bring about utilization of actionable personalized information. The creation of a virtual cloud of enormous masses of health-related data entries will be produced for each individual and analyzed as “big data.” It is interesting to note that almost 10 years ago, the United States National Institutes of Health initiated a program called “Big Data to Knowledge” (BD2K) to deal with the explosion of biomedical big data [44].

This so-called big data is defined as complex and voluminous data sets that cannot be processed via conventional data processing software because it contains exceptional variety, is collected in ever-increasing volumes, and arrives with increasing velocity [45]. This new field uses contemporary technology to analyze and systematically extract information from these massive data sets and is an emerging field in medicine [46]. Not surprisingly, dentistry, including periodontology, has begun to embrace this emerging field [47–49]. Early attempts to predict periodontitis in patients using big data analysis have demonstrated the potential of this technology [50]. However, there is considerable more work to be done before this becomes a mainstream part of patient care.

A central part of harnessing big data in periodontology will be to develop algorithms allowing correct diagnosis and classification/stratification of individuals to permit optimized prevention and treatment. This will require analyses of data at the population, individual, and molecular levels. For clinical practice, assessment of molecular and patient-level information will become increasingly important. For example, biological data gathered from various “omics” (e.g., genome, proteome, metabolome, degradome, microbiome, and inflammasome) could be used to complement traditional clinical examination measures (pocket depth, bleeding on probing, mobility, furcations, bone loss, etc.) and allostatic measures including socioeconomic status, lifestyle, and environment stressors. Thus, with the aggregate data, and sufficiently robust data analytic systems, it will be possible to differentiate categorize patients, based on their phenotype or symptoms, into stratified groups. This will allow individual disease trajectory to be forecast and personalized treatment to be implemented accordingly [14, 19].

## P4 Periodontics and treat-to-target concepts—bending the curve toward health

Over the past 40 years, we have begun to understand how inflammation and infection are interrelated. The end result of these interactions is considerable disease-related tissue destruction. More recently, the field of host modulation adjunctive therapy has begun to emerge whereby resolution of inflammation and return to tissue homeostasis has become of considerable importance [51]. Resolution of inflammation is focused toward bending the curve of inflammation-mediated tissue damage toward a health trajectory. Thus, a new paradigm for managing periodontitis based on controlling the inflammation has been presented [33]. It is based on the concept of resolving periodontal inflammation to levels that are compatible with periodontal health. The principal aim of this treatment philosophy is based on the medical model of “treat to target” and tries to shift the disease course from activity to remission. For many chronic medical conditions, the “treat to target” is a treatment strategy designed to define treatment targets based on validated disease severity scores [52]. This concept allows a disease to be considered in the context of its life course and uses specific clinical and biologic parameters as target endpoints in managing the pathology of the disease.

Adopting these principles in periodontology is consistent with the philosophy of P4 periodontics and is specifically aligned with changing the trajectory of the periodontitis disease course. It is designed to move the principal focus of treatment from anti-infective to modulation of inflammation. This does not disregard the important role of dental plaque and periodontal infection but refocuses attention on the central role of inflammation in critical nexus between infection and inflammation [35].

A number of driving principles for utilizing the treat-to-target concept, based on the 2014 recommendations of an international task force, for treating rheumatoid arthritis “to target” have been adopted for incorporation of this concept into periodontology (Table 3) [33, 53].

**Table 3:** Ten principles for adopting a “treat to target” approach for the management of periodontitis*

1. An initial target for treatment of periodontitis should be a state of clinical remission that then allows reconstructive and regenerative procedures to follow if necessary
2. Clinical remission will be defined as the absence/reduction of signs and symptoms of significant inflammatory disease activity that are responsible for the tissue damage associated with active periodontitis
3. While remission should be a clear target, based on available evidence low disease activity may be an acceptable alternative therapeutic goal, particularly in long-standing refractory disease
4. Until the desired treatment target is reached, therapies (mechanical, anti-inflammatory, and anti-infective) should be adjusted every 3–4 months
5. Measures of disease activity must be obtained and documented regularly, as frequently as 3–4 monthly for patients with high/moderate disease activity or less frequently (such as every 6–9 months for patients in sustained low disease activity or remission
6. The use of validated composite measures of diseases activity, which include periodontal assessments, is needed in routine clinical practice to guide treatment decisions
7. Structural changes and functional impairment should be considered when making clinical decisions (i.e., predisposing factors)
8. The desired treatment target should be maintained throughout the remaining course of the disease
9. The choice of the (composite) measure of disease activity and the level of target value will be influenced by consideration of co-morbidities, patient factors, drug-related risks, and microbiological profile
10. The patient has to be appropriately informed about the treatment target and the strategy planned to reach this target under the supervision of the periodontist

^*^Reproduced with permission from Bartold and Van Dyke[30]

**Fig. 6:**
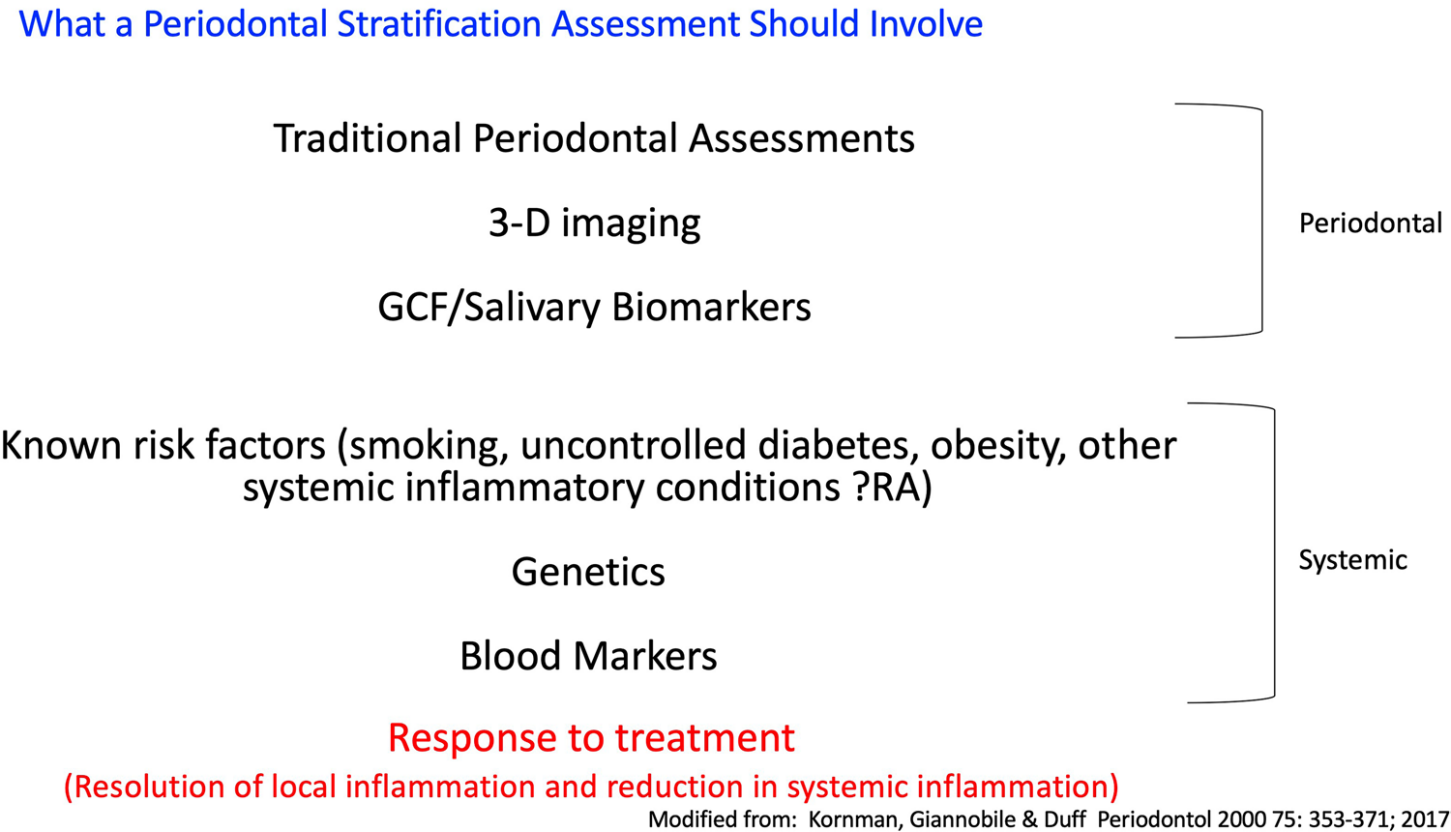
Patient disease stratification will incorporate both periodontal and systemic parameters as well as response to treatment. Modified with permission from Kornman et al. [22]

Using traditional periodontal diagnostic tools together with measures of local and systemic inflammation, a Periodontitis Disease Activity Score could be developed and based on the disease stratification alluded to earlier. Disease stratification will incorporate both periodontal and systemic parameters as well as response to treatment (Fig. 6). Thus, modifiable traditional markers, modifiable inflammatory markers, and modifiable systemic risk factors can be taken into account when considering an individual’s disease status (Table 4).

**Table 4:** Modifiable traditional markers, modifiable inflammatory markers, and modifiable systemic risk factors*

Modifiable traditional “markers”
• Pocket depth
• Bleeding on probing
• Predisposing factors
• Bacterial burden (plaque/ “pathogens”)
Modifiable inflammatory markers (local and systemic)
• PISA score
• CRP
• IL-1
• PGE2
• Oxidative stress
Modifiable systemic risk factors
• Smoking
• Diabetes
• Chronic inflammatory conditions
• Hormonal modifiers
• Diet

^*^Reproduced with permission from Bartold and Van Dyke[30]

Once collated, this information can be processed to determine the level of periodontal inflammation at both the local and systemic level to be used both a baseline recording as well as treatment target. In this model, defined clinical and biochemical data aligned to the host inflammatory response can be utilized to determine the success, or otherwise, of treatment. An interesting early attempt to do develop a Periodontal Summary Score has been used to stratify patients into classes of health, moderate disease, and advanced disease [54].

The Treat to Target model for the management of periodontitis is consistent with the P4 periodontics model in that it embraces disease personalization of the individual’s disease path, prediction of future disease experience, preventing disease by changing the life-course trajectory, and encourages participation by the patient to understand their treatment goals.

## The health continuum—repairing and regenerating damaged tissues

### Personalized periodontal regenerative medicine

In the late 1980s, periodontology entered the era of regenerative periodontics. Scientific developments since then have paved the way for considerable progress in periodontal regeneration using a combination of cell-based and tissue engineering concepts. Thus, rather than focusing only on resolving the accumulation of inflammation-mediated tissue damage, regenerative technologies aim to restore the tissues to their original architecture and function. To this end, tissue engineering may be particularly suited to periodontal regeneration as it facilitates the regeneration of tissue form and function through the implementation of supportive and bioactive scaffold structures [55].

The implementation of periodontal regenerative technologies is consistent with the P4 periodontics concept in that the ultimate goal of periodontal treatment is to restore the periodontium to its original architecture and function. This is particularly relevant to advanced disease (stage 3 and 4), where even following disease remission, significant functional and aesthetic challenges remain that negatively impact an individual’s quality of life. Together, the concepts of P4 periodontics and regenerative periodontics are complimentary and will serve to restore damaged periodontal structures to health in both their structure and function by directing crucial cellular and molecular processes needed for restoration of the tissues to their original integrity.

### Current and future developments in personalized/precision periodontal regenerative medicine

The development of advanced biomaterials, featuring customized material chemistry, device fabrication, biofunctionalization, and/or patient data analysis, has considerable potential to transform precision medicine [56].

Nevertheless, the clinical implementation of implantable devices in personalized/precision medicine is challenging due to the need to understand the patient–device interactions in sufficient detail to inform design variables that can influence biological events at the level of the individual patient. Arguably, the development of customized highly porous biodegradable scaffolds utilizing medical-grade biomaterials currently offers the most promising prospects for clinical translation of personalized/precision implantable devices.

### Customized scaffold-guided tissue regeneration (S-GTR)

The site-specific nature of periodontal destruction means that the pattern of tissue loss in periodontitis is unique to each individual. Recent developments in advanced imaging and manufacturing, especially additive manufacturing such as 3D printing, provide the opportunity to fabricate personalized regenerative treatments to individuals via customized scaffolds that can facilitate the regeneration of specific defect geometries [57]. As an additive manufacturing technology utilizing a layer-by-layer material deposition, 3D printing is particularly suitable for the fabrication of tissue engineering scaffolds as it imparts porosity to the scaffold [58]. This porosity is essential for tissue infiltration within the defect, and in particular for allowing vascularization that is essential for regeneration.

A key clinical consideration for periodontal regeneration is the provision and maintenance of space for tissue ingrowth into a defect site and control over the type of tissue that repopulates the defect. Indeed, this forms the basis of the “guided tissue regeneration (GTR)” concept, which is a widely adopted clinical modality for periodontal regeneration based on the principle of using an occlusive membrane to provide space for, an exert control over, the type of tissue re-populating a healing periodontal defect. In this context, the use of personalized scaffolds is congruent with the concept of GTR because additive manufacturing technologies, such as 3D printing, can be used to form porous and volumetrically stable scaffolds that provide space and control over the healing in a defect. Indeed, this “scaffold-guided bone regeneration (S-GBR) approach” is particularly suitable for periodontal regeneration, where the creation and maintenance of space for dimensionally stable tissue maturation are critical. Furthermore, control over the internal structures of the scaffolds allows the creation of multi-phasic scaffolds that permit temporo-spatial control over the healing within the defect [59], something the membrane-based GTR approaches are unable to achieve.

Proof of principle of the viability of this approach has been demonstrated via the clinical use of a customized 3D-printed medical-grade scaffold for the regeneration of an advanced periodontal defect [60]. While the initial integration of the scaffold was favorable, with good clinical outcomes at 12 months post-healing, parts of the scaffold were exfoliated after 13 months. The reason for the suboptimal outcome is unclear, but it is possible that the relatively low porosity of the PCL scaffold, which is inherently slowly resorbing, compromised the long-term integration of the implant with the surrounding periodontal tissues. This highlights the importance of the scaffold porosity, with a highly porous scaffold necessary for tissue integration in the oral environment, which has inherently challenging conditions for wound healing.

### Custom scaffold fabrication from medical-grade polymer

Various materials can be utilized for custom scaffold fabrication, including ceramics, polymers, composites, and even resorbable metals [61]. However, in the context of clinical translation, key considerations are the cost, clinical handling, regulatory status, and ease of manufacturing. In this regard, medical-grade polymers, such as polycaprolactone (PCL), provide attractive properties including cost-effectiveness, printability, ease of surgical fixation, resorbability, and existing regulatory approval for clinical use [62].

**Fig. 7:**
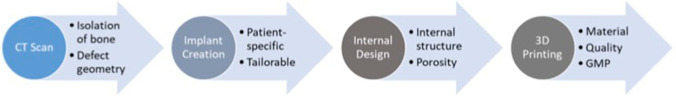
Workflow of customized scaffold design and fabrication, showing the different stages involving digital data acquisition, implant creation, internal design/porosity, and 3D printing

Highly porous custom medical-grade PCL scaffolds using 3D printing can be produced by implementing a published workflow (Fig. 7) from CT data [58]. First, CT scan data is imported into medical 3D image-based engineering software, such as Materialise Mimics (Materialise GmbH, Belgium), and a mask is created using a contrast threshold to isolate the bony tissues. The mask is then cropped to isolate the region of interest and adjacent tissues, and any noise from metallic implants is separated from the patient geometry by mask erasing. The mask is then converted into a continuous 3D model file and transferred to a computer-aided design (CAD) software such as Materialise 3-matic (Materialise GmbH). The CAD software uses the imported patient 3D defect model to guide the formation of a custom implant template design that precisely replicates the geometry of the periodontal defect. This 3D defect template model is exported as an STL file for fabrication by an extrusion 3D printer such as EnvisionTEC 3D-Bioplotter (EnvisionTEC GmbH). The 3D printer proprietary software (in this case, VisualMachines, EnvisionTEC GmbH) is used to design the internal features of the scaffold that impart the porosity. A highly porous (> 85%) scaffold is then printed using a medical-grade polymer, such as polycaprolactone (PCL), using optimized parameters [63]. For clinical translation, the fabrication processes should be defined by good manufacturing practice (GMP) documentation.

An important consideration in the clinical application of medical-grade polymeric biomaterials, which generally have reduced bioactivity, is the requirement to impart bioactivity via the use of bioactive molecules. In this regard, a practical consideration is the utilization of strategies with low regulatory barriers to clinical implementation, such as “point of care” functionalization with commercially available products or autologous preparations. In the context of periodontal regeneration, such “point-of-care” bedside biofunctionalization can be achieved by combining the scaffold with commercially available bioactive molecules (e.g., enamel matrix derivative (EMD) [64], fibroblast growth factor (FGF-2) [65], platelet-derived growth factor (PDGF) [66]), or possibly with more cost-effective autologous formulations such as platelet concentrates (e.g., platelet-rich fibrin (PRF) [67], platelet-rich plasma (PRP) [68]). It should, however, be noted that although autologous blood-derived products show some promising outcomes in promoting periodontal regeneration, their efficacy still require further validation in clinical trials, and do have the inherent disadvantage of compositional variability depending on both the source (individual patient) and the preparation method.

### The future beyond custom scaffolds: precision biomaterials for personized medicine

As alluded to earlier in this review, advances in high-throughput “omics” and microbiome technologies have provided new insights into the heterogeneity of disease and response to treatments [69], which in turn presents an opportunity for using this metadata to enable personalized treatments to be administered via an implanted device. Indeed, precision biomaterials can play an important role as cell delivery vehicles in novel patient-specific cellular therapies developed via editing and cellular reprogramming [70].

Furthermore, in addition to biofabrication advances that permit precision biomaterial-based devices to be manufactured across various length scales (from macro to micro to nano), novel developments in material chemistry allow for modulation of biofunctionality and controlled responses to local analytes or environmental conditions [71]. In this context, biomaterial design, beyond geometrical customization, could impart important biological characteristics that confer the ability to monitor disease and facilitate triggered responsiveness to local stimuli, which can be individualized for enhanced precision based on “omics” metadata that characterizes an individual’s response to different disease states.

Thus, future personalized treatments may utilize a combination of multifunctional biomaterials that act in a precise manner. For example, in the context of periodontal treatment, a construct inserted to initially facilitate regeneration may also be utilized to enhance the long-term maintenance of health by being functionalized with the ability to recognize specific external stimuli (e.g., threshold inflammatory burden or bacterial load) and respond to this altered environment by releasing one or more pay loads (such as antimicrobial or anti-inflammatory agents). Central to this approach is a deep understanding of the host–implant interaction that is sufficiently detailed to facilitate the coordination of this complex series of biological events at the level of the individual.

An example of this type of approach is the evolution of a newly emerged branch of regenerative medicine called “rejuvenation medicine” whereby rejuvenation biotechnologies will utilize the advances of regenerative medicine to assist in managing the damage of aging [72]. Thus, through the principles of P4 medicine to identify and manage the accumulating effects of aging, rejuvenation technologies will target the removal, restitution, or replacement of tissues affected by age-related cell and molecular decline. For periodontology, this means that treatments will begin to focus on not only reducing the effects of ongoing degeneration but restoration of the periodontal structures to a healthier structure and function through the rejuvenation of the cells and tissues of the periodontium. Thus, treatments will refocus from risk management to regenerative solutions to manage the effects of disease and aging. Of course, these approaches are not without their obstacles with some investigators questioning the likelihood of success with the emerging field of rejuvenation biotechnologies [73].

## Concluding comments

Traditionally, the management of plaque-associated periodontitis has been reactive to its etiology (plaque) and consequences (tissue damage). By adopting the concepts of P4 periodontics, plaque-associated periodontitis can be managed proactively to achieve the goal of healthy aging using a novel holistic management strategy. The underlying tenets of P4 periodontics, as for P4 medicine, are prediction, prevention, personalization, and participation. These four pillars enable both oral health care workers and their patients to work together to encourage health and well-being throughout the life course of disease experience. It embraces the concept of identifying and managing the continuum from health to advanced disease with the goal of returning the disease path to a specific target of health or remission. This will involve acquiring wide sets of data across the health spectrum at an early life-course stage. By using analytic systems–based technologies and multi-partner interdisciplinary proactive interventions, together with active patient participation, an overarching healthcare plan can be devised, implemented, and monitored. This will span early prevention and intervention through to late-stage reversal of the disease trajectory and regenerative procedures consistent with reconstructing damaged tissues and restoring health.
